# Bone Metastasis in Estrogen Receptor-Positive Breast Cancer: Molecular Insights and Therapeutic Advances

**DOI:** 10.3390/ijms27020785

**Published:** 2026-01-13

**Authors:** Zhuoran Huang, Yi Wu, Yanshu Li

**Affiliations:** 1 School of Medicine, China Medical University, Shenyang 110122, China; 2023318218@cmu.edu.cn; 2 Department of Pathogenic Biology, Shenyang Medical College, Shenyang 110034, China; 3 Department of Cell Biology, Key Laboratory of Cell Biology, Ministry of Public Health, and Key Laboratory of Medical Cell Biology, Ministry of Education, China Medical University, Shenyang 110122, China

**Keywords:** estrogen receptor-positive breast cancer, bone metastasis, endocrine resistance, CDK4/6 inhibitors, signal transduction, tumor microenvironment, targeted therapy, precision medicine

## Abstract

Estrogen receptor-positive (ER^+^) breast cancer represents the most prevalent molecular subtype of breast cancer, characterized by hormone-dependent growth, relatively indolent progression, and a pronounced tendency to metastasize to bone. While endocrine therapies remain the cornerstone of treatment, a significant proportion of ER^+^ tumors eventually develop resistance, culminating in distant metastases—most frequently to the bone. Bone metastasis substantially compromises patient survival and quality of life, highlighting the critical need to elucidate its molecular underpinnings. Recent multi-omics and mechanistic studies have shed light on the complex interplay between tumor-intrinsic signaling pathways, such as dysregulated ER signaling, PI3K/AKT/mTOR, TGF-β, and Hippo pathways, and the bone microenvironment, including osteoclast activation, immune suppression, and stromal remodeling. This review systematically summarizes the current understanding of the molecular mechanisms driving bone metastasis in ER^+^ breast cancer, with a particular focus on tumor–bone microenvironment crosstalk and key regulatory pathways. Additionally, we discuss recent advances in therapeutic strategies, encompassing next-generation endocrine therapies, CDK4/6 inhibitors, bone-targeted agents, and pathway-specific inhibitors. Together, these insights pave the way for more effective and personalized interventions against ER^+^ breast cancer with bone involvement.

## 1. Introduction

According to data released by the World Health Organization in 2024, breast cancer remains the most diagnosed malignancy among women in 157 out of 185 surveyed countries. The incidence of breast cancer is 23.8% in females. Despite continuous progress in diagnosis and treatment, a significant proportion of patients present with no identifiable risk factors and continue to face a high mortality rate of 15.4% [1]. Both direct and indirect medical expenditures are significant burdens for most patients during the diagnosis and treatment process of breast cancer; meanwhile, increasing societal consequences cannot be neglected [2]. Among the leading causes of breast cancer–related death, bone metastasis is particularly critical [3]. Breast cancer–derived bone metastases commonly affect the sternum, ribs, vertebrae, pelvis, and femur, significantly compromising both the physical function and psychological well-being of patients [4]. Epidemiological studies indicate that the five-year overall survival rate for patients with bone metastasis is less than one-quarter that of those without skeletal involvement [5,6]. The bone microenvironment harbors a local estrogen axis, in which estrogen is known to promote the progression of primary breast tumors [7]. Among the key prognostic indicators, hormone receptor status plays a decisive role in determining clinical outcomes. Estrogen receptor (ER)-positive breast cancer, which comprises about 70% of diagnosed breast carcinoma, represents the most prevalent subtype and demonstrates a marked predilection for bone metastasis [8]. Compared to ER-negative tumors, ER-positive breast cancers tend to exhibit a prolonged latency period (often spanning 8–10 years) before the emergence of overt skeletal metastasis [9,10]. Without vigilant monitoring, this dormancy may result in missed opportunities for early intervention.

Breast cancer is subtyped based on ER, progesterone receptor (PR) and Human epidermal growth factor receptor 2 (HER2) status. Furthermore, 70% of patients with primary breast tumors experience ER^+^, and ER^+^ breast cancer has a high tendency to metastasize to distant sites; in particular, bone metastasis is most frequently observed, which is a major cause of breast cancer-associated morbidity and mortality [11,12]. ER^+^ tumors depend on estrogen signaling for their growth and proliferation, making endocrine therapies, including selective estrogen receptor modulators (SERMs), selective estrogen receptor downregulators/degraders (SERDs), and aromatase inhibitors (AIs), the cornerstone of treatment. However, despite initial therapeutic success, many ER^+^ breast cancer patients develop metastatic disease, with bone being the most frequent site of metastasis [13,14,15]. It has not been clearly revealed how bone offers a better microenvironment for ER^+^ tumor cell survival. Before forming secondary tumors of the bone, cancer cells display a superior invasive capacity, immune escape ability, and chemotherapy resistance gene expression and seek environments that support dormancy [16].

In this review, we provide a comprehensive overview of bone metastasis in ER^+^ breast cancer, with a focus on genetic alterations, key signaling pathways, and tumor–microenvironment interactions that facilitate metastatic progression and skeletal colonization. Notably, we describe the development history of breast cancer bone metastasis from descriptive observations to mechanism-based therapies. Given the high bone tropism of ER^+^ tumors, elucidating the molecular underpinnings of this process is essential for improving patient outcomes and developing effective targeted therapies. Recent studies have identified a growing number of genes and regulatory networks involved in the initiation, progression, and bone-specific colonization of tumor cells. In the following sections, we summarize these critical molecular drivers and their functional roles in the metastatic cascade of ER^+^ breast cancer.

## 2. Historical Development of Research on ER^+^ Breast Cancer Bone Metastasis

**Figure 1:**
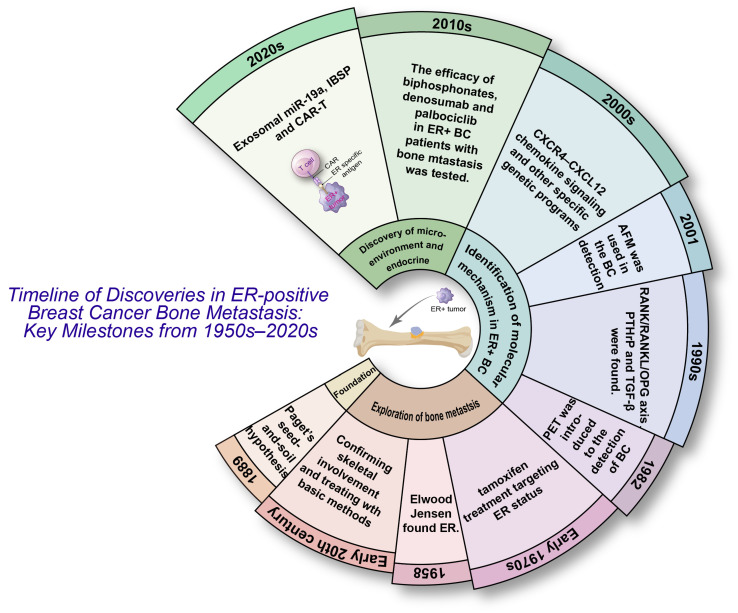
Timeline of major discoveries in ER^+^ breast cancer bone metastasis. The figure outlines key molecular and therapeutic milestones of ER^+^ breast cancer bone metastasis. Major advances include the identification of ER signaling, bone-tropic mechanisms such as PTHrP and RANKL–RANK, and the development of endocrine-targeted therapies.

The scientific understanding of ER^+^ breast cancer bone metastasis has evolved through more than a century of observations, mechanistic discoveries, and therapeutic advances (Figure 1). The earliest foundation was laid by Paget’s 1889 seed-and-soil hypothesis, which proposed that the marked tropism of breast cancer was bone [17]. Throughout the early 20th century, several studies repeatedly confirmed the predominance of skeletal involvement in breast cancer and tried some basic methods for treatment, though the biological basis remained undefined [18,19].

A major conceptual shift occurred with the discovery of the estrogen receptor in 1958, establishing hormonal dependence as a defining biological feature of a large subset of breast cancers. ER status became clinically actionable with the introduction of tamoxifen, which was the first targeted endocrine therapy in the early 1970s [20]. Over subsequent decades, clinicopathologic correlations demonstrated that ER^+^ tumors show a strong propensity for bone-dominant recurrence, often after prolonged disease-free intervals. The recognition of latent metastatic dormancy in ER^+^ disease further explained the epidemiological pattern of late skeletal relapse.

From the 1990s onward, research shifted toward defining molecular determinants of breast cancer osteotropism. A breakthrough was the osteoclast-driven vicious cycle model. Breast cancer cells secrete PTHrP, inducing osteoblast-derived RANKL and activating osteoclasts. Bone resorption then releases matrix-embedded TGF-β, which enhances tumor invasion, PTHrP expression, and metastatic expansion—thus forming a self-reinforcing loop. Concurrently, the RANK/RANKL/OPG axis was identified as the core regulator of osteoclast genesis, linking bone remodeling directly to tumor growth [21].

Parallel work uncovered tumor-intrinsic drivers of bone tropism. CXCR4–CXCL12 chemokine signaling was shown to mediate tumor homing to bone marrow niches [22]. Kang et al. identified a distinct bone-metastasis gene signature, establishing that metastatic osteotropism is encoded by specialized genetic programs rather than stochastic dissemination [23]. These signatures later proved predictive of skeletal relapse across clinical cohorts, particularly in ER^+^ patients.

More recent studies have revealed mechanisms unique to ER^+^ bone metastasis. ER^+^ tumor cells exhibit pronounced long-term dormancy within bone marrow niches, remaining quiescent for years until reactivated by inflammatory or microenvironmental cues. Furthermore, the bone microenvironment promotes endocrine resistance. Exosomal miR-19a and IBSP was identified as key ER^+^-specific metastasis drivers, providing the first evidence of coordinated exosomal and matricellular control of ER^+^ osteolytic disease [12].

Therapeutic advances have accompanied these mechanistic insights. Bisphosphonates and denosumab, targeting osteoclast activity via inhibition of farnesyl pyrophosphate synthase and neutralization of RANKL, respectively, became standard treatments for skeletal metastases [24]. Large meta-analyses demonstrated that adjuvant bisphosphonates reduce bone recurrence and mortality in postmenopausal early breast cancer [25]. The introduction of CDK4/6 inhibitors has further improved systemic control [26].

These historical milestones illustrate a progression from observational pathology to precise mechanistic understanding and targeted therapy, establishing ER^+^ bone metastasis as a biologically distinct entity shaped by hormone signaling, tumor–bone crosstalk, and niche-driven evolution.

## 3. Molecular Drivers of Bone Metastasis in ER^+^ Breast Cancer

Bone metastasis in ER^+^ breast cancer is driven by a complex interplay of genetic factors that modulate tumor cell behavior and the bone microenvironment. These regulatory genes influence multiple steps of the metastatic cascade, including dormancy, immune evasion, osteoclast activation, and colonization through their interaction with hormone signaling and other regulatory networks. To better characterize these factors, the genes implicated in ER^+^ skeletal metastasis can be broadly categorized into two groups: Hormone-dependent genes, which are directly or indirectly regulated by the ER pathway, and hormone-independent genes, which function outside of classical ER signaling but still contribute to metastatic potential via parallel pathways. This classification provides a clearer framework for understanding the molecular mechanisms underlying skeletal colonization in ER^+^ breast cancer and may help identify more targeted therapeutic strategies.

### 3.1. Hormone-Dependent Genes

Hormone-dependent genes exert their pro-metastatic roles by acting downstream of estrogen signaling. They participate in epithelial–mesenchymal transition (EMT) induction, dormancy regulation, immune escape, and osteoclast-mediated bone remodeling. Below, we discuss representative genes in this category with known links to ER signaling and bone metastasis biology.

#### 3.1.1. Signal Peptide-CUB-EGF-like Domain-Containing Protein 2 (SCUBE2)

SCUBE2 is a secretory protein directly regulated by estrogen receptor alpha (ERα), as demonstrated by chromatin immunoprecipitation (ChIP) and reporter assays in ER^+^ breast cancer cells. SCUBE2 plays a dual role in breast cancer progression. On one hand, it acts as a tumor suppressor by inhibiting β-catenin signaling, and its expression correlates with improved prognosis in ER-positive patients [27,28]. On the other hand, its overexpression in luminal A/B subtypes facilitates osteotropic metastasis. Mechanistically, SCUBE2 enhances Hedgehog (HH) signaling by facilitating Sonic Hedgehog (SHH) release and interaction with PTCH1 on osteoblasts [29,30,31]. This promotes endochondral ossification and osteoblast differentiation, thereby priming the bone niche for colonization. More critically, SCUBE2-mediated SHH signaling induces collagen deposition by osteoblasts, which binds to LAIR1 on natural killer (NK) cells and suppresses immune surveillance, enabling metastatic escape in the bone microenvironment [29]. Furthermore, SCUBE2 expression contributes to Notch pathway activation and EMT in breast cancer stem-like cells, increasing their invasiveness and bone-tropic capacity [32].

#### 3.1.2. p21-Activated Kinase 4 (PAK 4)

Like SCUBE2, PAK4 is transcriptionally responsive to ER signaling, and participates in both dormancy regulation and bone destruction. PAK4 has emerged as a potential therapeutic target due to its multifaceted role in osteolytic metastasis, endocrine resistance, and tumor progression [33,34]. In the classical estrogen signaling pathway, 17β-estradiol (E2) binds to estrogen receptors (ERα and ERβ), inducing dimerization and interaction with estrogen response elements (EREs) on DNA via coregulatory proteins [35,36]. Upon E2 stimulation, PAK4 interacts with ERα and translocates from the cytoplasm into the nucleus (nPAK4) in ER^+^ breast cancer cells. In the nucleus, nPAK4 suppresses the expression of leukemia inhibitory factor receptor (LIFR), a key ERα target gene responsible for maintaining the dormant state of disseminated tumor cells (DTCs) in the bone marrow niche [33]. LIFR signaling, triggered by ligands such as LIF, OSM, and CNTF, activates pathways including JAK/STAT3, AKT/mTOR, and Hippo/YAP, which collectively support dormancy [37,38,39]. Loss of LIFR expression by nPAK4 reactivates dormant cells, promoting migration and colonization. Beyond dormancy escape, nPAK4 promotes osteoclast-mediated bone degradation. It phosphorylates RUNX1 at threonine-207, resulting in nuclear export and recruitment of the SIN3A/HDAC1 transcriptional repressor complex. In conjunction with PRMT1, phosphorylated RUNX1 (p-RUNX1) activates Jagged1 and Parathyroid hormone-related protein (PTHrP), two master regulators of osteoclast differentiation and osteolysis via Notch and PTHrP signaling, respectively.

#### 3.1.3. TOX High Mobility Group Box Family Member 3 (TOX3)

TOX3 is a transcriptional regulator predominantly expressed in ER-positive breast epithelial cells and is modulated by ER signaling. It plays a dual role in breast cancer, acting as both a tumor suppressor and a pro-metastatic factor depending on the cellular context. During early tumorigenesis, TOX3 is expressed in breast epithelial progenitor cells and has been linked to tumor initiation. Functionally, TOX3 regulates the expression of multiple genes involved in proliferation and metastasis, including TFF1 and CXCR4, which are known as ER target genes [40]. Paradoxically, while some studies associate TOX3 with tumor suppression, elevated TOX3 expression has also been linked to poor prognosis and an increased risk of bone metastasis, particularly in ER-positive breast cancer patients [41,42].

#### 3.1.4. Nuclear Receptor Interacting Protein 1 (NRIP1)

NRIP1 encodes a nuclear receptor co-regulator that modulates ER signaling and inhibits mitotic activity by interacting with FHL1 [43]. Functioning as a cofactor within the endoplasmic reticulum, NRIP1 plays a key role in controlling ER-positive breast cancer cell proliferation. Genetic studies have identified rs28323093, a variant located downstream of NRIP1, as being associated with ER-positive breast cancer, likely through altered NRIP1 expression [44]. These findings suggest that NRIP1 acts as a hormone-dependent modulator, linking genetic variation to dysregulation of ER signaling and contributing to tumor progression.

In addition to the genetic alterations that predispose ER^+^ breast cancer cells to skeletal colonization, growing evidence indicates that these genomic drivers exert their pro-metastatic effects through intricate signaling networks. These pathways regulate critical cellular behaviors, including proliferation, migration, immune evasion, and resistance to apoptosis, while concurrently remodeling the bone microenvironment to support metastatic engraftment and progression. The subsequent section delineates the principal signaling cascades such as transforming growth factor-beta (TGF-β), BMP-SMAD, Hippo, and ERα-Src-p190 RhoGAP that govern the pathophysiology of ER^+^ breast cancer bone metastasis and elucidates the pivotal regulatory proteins that serve as integrative nodes within these interconnected networks.

### 3.2. Hormone-Independent Genes

Studies have shown that bone morphogenetic protein (BMP) genes are highly expressed in ER-positive breast cancer, and BMP proteins can promote angiogenesis by enhancing EMT [45]. They regulate the PI3K/AKT pathway and Fam20C, control osteoclast differentiation, and cooperate with TGF-β proteins to induce bone metastasis and colonization of breast cancer cells [46,47,48,49]. For some BMP receptors, ACVRL1 and TGFBR2 may be closely related to angiogenesis. When TGFBR2 is downregulated, miR-204 can exert an inhibitory effect on tumor angiogenesis [50]. Overall, BMP signaling contributes to bone metastasis in ER-positive breast cancer by promoting angiogenesis, EMT, and osteoclast differentiation through coordination with pathways such as PI3K/AKT, Fam20C, and TGF-β. These findings highlight that, beyond classical hormone-dependent mechanisms, ER-positive breast cancer progression and bone metastasis are also driven by hormone-independent pathways. These include BMPs, TGF-β, CXCR4, PTHrP, and other molecular mediators that are not directly regulated by ER but are essential for metastatic dissemination. The integration of these signaling axes underscores the complexity of metastatic progression and reveals potential therapeutic targets beyond endocrine manipulation.

#### 3.2.1. Torsin Family 1 Member B (TOR1B)

TOR1B gene plays a crucial role in multiple cellular processes, including protein processing and transport within the endoplasmic reticulum [51]. Elevated expression of TOR1B has been associated with enhanced bone metastasis and poor prognosis in breast cancer patients [52]. Evidence suggests that HIF1A may regulate TOR1B expression in basal-like breast cancer cells, linking TOR1B to hypoxia-related signaling pathways. Notably, TOR1B knockdown has been shown to impair cellular metabolic activity and increase apoptotic cell death, highlighting its role in promoting tumor cell survival and adaptation under stress conditions [53]. Collectively, the data highlight TOR1B as a potential driver of metastasis and cellular stress tolerance in breast cancer, offering a promising target for therapeutic intervention.

#### 3.2.2. PTHrP

PTHrP plays an essential physiological role in normal breast development and skeletal homeostasis, particularly during lactation, where it mediates calcium mobilization from bone to support milk production [54,55]. However, in the context of breast cancer, tumor-derived PTHrP acquires pathological functions. It can induce humoral hypercalcemia of malignancy and facilitate osteolytic bone metastasis by stimulating osteoclast differentiation and bone resorption. Moreover, PTHrP has been shown to enhance the survival of tumor cells under apoptotic stress, thereby promoting tumor cell proliferation and metastatic potential [54,56,57]. By disrupting normal bone remodeling and creating a favorable microenvironment for cancer cell colonization, PTHrP contributes significantly to the vicious cycle of bone metastasis in breast cancer. Overall, PTHrP serves as both a mediator of physiological calcium homeostasis and a pathological driver of breast cancer progression and bone metastatic colonization, highlighting its potential as a therapeutic target in metastatic breast cancer.

Additionally, Dilara, C. et al. identified 15 differentially expressed genes by comparing breast cancer bone metastases with non-bone metastatic tumors. Among these, NAT1 (involved in metabolic processes), PH-4 (related to redox balance), and BBS1 (associated with protein transport) were upregulated, while PALM2, STEAP3, MFAP3L, NUP155, C6ORF167, NIP7, C16ORF61, PGBD5, KCNS1, SFT2D2, ATL2, and APOBEC3B were downregulated [58]. These genes are thought to contribute to modifying the tumor microenvironment, facilitating bone-specific colonization by breast cancer cells. In a complementary study, Kang et al. reported a bone metastasis gene signature consisting of 102 genes, which may further refine the understanding of bone metastatic mechanisms and support targeted gene editing or therapeutic interventions [23]. Together, these gene sets provide valuable insights into the molecular alterations associated with breast cancer bone metastasis and may serve as potential biomarkers or therapeutic targets.

In summary, ER-positive breast cancer bone metastasis is orchestrated by both hormone-dependent and -independent genetic mechanisms. ER-regulated genes such as SCUBE2, PAK4, TOX3, and NRIP1 exert pivotal influence through hormonal signaling, while non-hormonal drivers like TOR1B and PTHrP promote metastatic survival under metabolic or immunologic stress (Table 1). Understanding these distinct yet interconnected axes may pave the way for precise therapeutic targeting bone-metastatic breast cancer.

## 4. Networked Signaling Pathways Underlying Bone Metastasis in ER-Positive Breast Cancer

**Figure 2:**
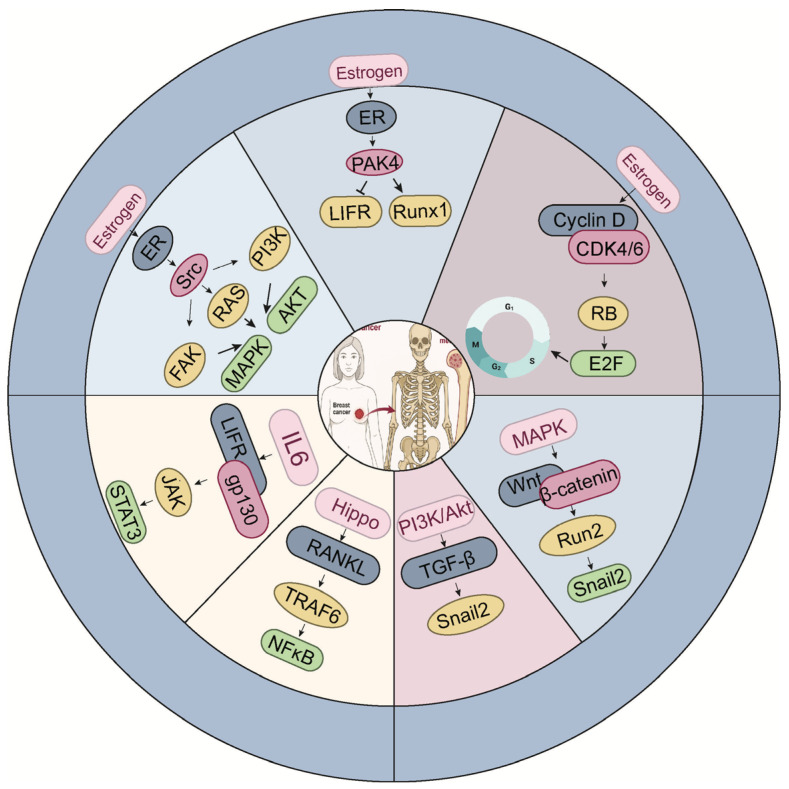
Estrogen-dependent and -independent signaling pathways in breast cancer bone metastasis. This schematic integrates major molecular axes that drive tumor progression and osteotropism, distinguishing estrogen-dependent pathways—ER–FAK–RAS–MAPK–PI3K–Akt promoting survival and migration, ER–PAK4–LIFR–Runx1 facilitating osteotropic adaptation, and Cyclin D–CDK4/6–RB–E2F driving cell cycle progression—from estrogen-independent pathways, including MAPK/PI3K–Wnt/β-catenin–Runx2–Snail2 enhancing osteomimicry and EMT, TGF-β–Snail2 reinforcing EMT plasticity, and IL6–LIFR–gp130–JAK–STAT3–RANKL–TRAF6–NFκB activating osteoclast-mediated bone resorption; collectively, these interconnected signals highlight how both estrogen-dependent and independent mechanisms remodel the bone microenvironment to facilitate metastatic dissemination of breast cancer cells.

**Figure 3:**
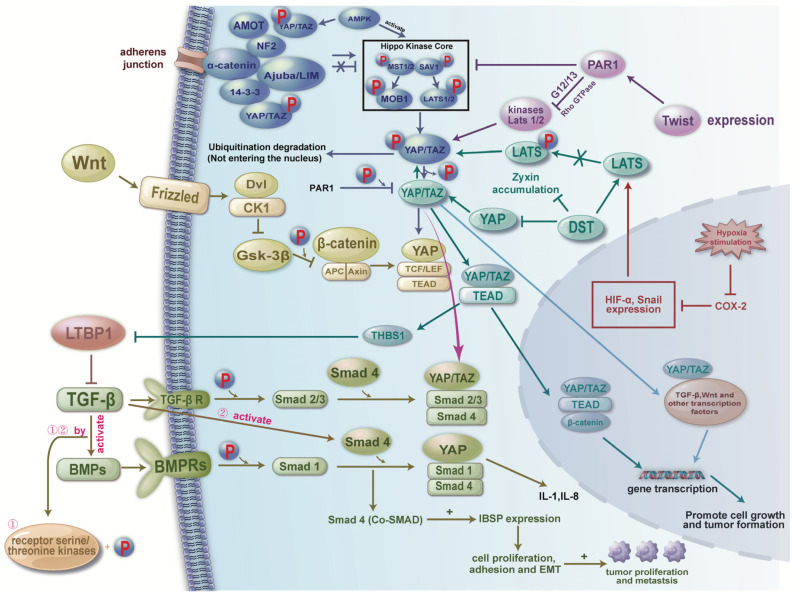
Cooperative signaling pathways orchestrating breast cancer bone metastasis. This schematic highlights the convergence of multiple intracellular networks—including Wnt/β-catenin, TGF-β/Smad, and Hippo/YAP–TAZ pathways—together with additional regulators such as PAR1, Twist, COX-2, HIF-α, and adherens junction proteins. These pathways collectively modulate gene transcription, cell proliferation, epithelial–mesenchymal transition (EMT), and tumor–microenvironment interactions, thereby remodeling bone homeostasis and facilitating metastatic colonization. The figure emphasizes that bone metastasis is not driven by a single axis but arises from the synergistic regulation of diverse signaling modules that integrate proliferative, migratory, and osteotropic programs. Arrows indicate activation or promotion, whereas blunt-ended lines indicate inhibition. “P” denotes phosphorylation. Numbers ①② indicate different signaling routes downstream of TGF-β/BMP receptors.

The establishment of bone metastasis in ER^+^ breast cancer is not solely determined by tumor-intrinsic properties, but also by dynamic interactions with the bone microenvironment, which is tightly regulated by complex signaling networks (Figure 2). To align with the mechanistic description in this section, Figure 2 organizes the signaling network into an estrogen-dependent domain (the upper part) and an estrogen-independent domain (the lower part), providing a structured overview of the dual regulatory inputs governing metastatic behavior in ER^+^ disease. Among these, osteoblast differentiation from mesenchymal stem cells plays a pivotal role in shaping the skeletal niche. Multiple signaling molecules, such as TGF-β, endothelin-1 (ET-1), fibroblast growth factors (FGFs), platelet-derived growth factors (PDGFs), and bone morphogenetic proteins (BMPs), orchestrate this process by activating the master osteogenic transcription factor Runx2, which governs osteoblast lineage commitment and bone matrix production [59] (Figure 3). While these pathways are essential for physiological bone homeostasis, accumulating evidence suggests that they are frequently co-opted by ER-positive breast cancer cells to facilitate metastatic colonization and survival within the bone. In this section, we delineate the major signaling axes that contribute to ER-positive breast cancer bone metastasis, emphasizing how their integration promotes tumor-bone interactions, osteotropism, and therapeutic resistance. We visually integrate these signaling routes and show how Hippo–YAP/TAZ, TGF-β, and Wnt pathways collectively influence tumor cell plasticity and metastatic behavior in ER^+^ disease (Figure 3).

### 4.1. TGF-β Signaling Pathway

TGF-β signaling pathway plays a pivotal role in promoting bone metastasis in breast cancer, particularly by driving osteolytic processes. TGF-β has been shown to induce the expression of key osteolytic genes, such as interleukin-1 (IL-1) and connective tissue growth factor (CTGF), which are highly expressed in osteolytic lesions [23]. Moreover, TGF-β enhances the expression of PTHrP, interleukin-11 (IL-11), receptor activator of nuclear factor kappa-B ligand (RANKL), and matrix metalloproteinases (MMPs), thereby stimulating osteoclast activity and accelerating bone resorption [60,61,62]. Interestingly, the tumor suppressor gene ITIH5 has been shown to modulate TGF-β superfamily signaling, thereby attenuating the metastatic potential of breast cancer cells [63]. In the bone microenvironment, particularly within ER-positive osteoblasts, intracellular second messenger cascades are frequently upregulated, suggesting potential crosstalk between ER signaling and TGF-β activation [64]. Recent studies have further highlighted the EZH2–integrin β1–FAK axis as a critical enhancer of TGF-β signaling during bone metastasis. EZH2 transcriptionally upregulates integrin β1, which in turn activates focal adhesion kinase (FAK). This leads to tyrosine phosphorylation of TGF-β receptor I (TGFβRI), thereby increasing its affinity for TGF-β receptor II (TGFβRII) and amplifying downstream TGF-β signaling [65]. However, it is important to note that the anti-metastatic effects of EZH2 inhibitors are not universal and appear to be highly context dependent. While EZH2 inhibition may effectively suppress tumor dissemination to certain organs, such as the lung, paradoxically, it can promote metastatic colonization in the bone. These findings highlight the necessity of organ-specific considerations and careful therapeutic stratification when applying EZH2-targeted interventions in clinical settings [66,67].

### 4.2. BMP-SMAD Signaling Pathway

BMP-SMAD signaling cascade is a fundamental regulator of osteogenic differentiation and bone homeostasis, and it plays a pivotal role in the initiation and progression of breast cancer bone metastasis. BMPs, members of the TGF-β superfamily, bind to serine/threonine kinase receptors (BMPR-I/II) on the cell surface, leading to phosphorylation of receptor-regulated SMADs (R-SMADs, primarily SMAD1/5/8). These R-SMADs subsequently form heteromeric complexes with SMAD4 and translocate into the nucleus, where they regulate the transcription of target genes involved in extracellular matrix remodeling, osteoclast activation, and tumor-bone microenvironment crosstalk. Activation of the BMP–SMAD axis has been shown to synergize with TGF-β1 signaling during metastatic colonization. TGF-β1 can potentiate the expression of osteolytic cytokines such as IL-11 and interleukin-8 (IL-8) through the SMAD signaling cascade, thereby enhancing osteoclast differentiation and bone resorption [68]. In addition, integrin-binding sialoprotein (IBSP), a major bone matrix-associated glycoprotein and SMAD-responsive gene, is frequently overexpressed in bone-metastatic breast cancer cells. IBSP facilitates the anchorage and colonization of disseminated tumor cells (DTCs) within the mineralized bone matrix, reinforcing the vicious cycle of tumor-induced bone destruction [69].

Interestingly, the role of BMP ligands is context-dependent in ER-positive breast cancer. Elevated expression levels of BMP6 and BMP7 have been associated with enhanced anti-tumor immune responses, as these ligands increase cytotoxic immune cell infiltration and augment their effector functions, correlating with improved clinical prognosis in ER-positive subtypes [70]. This suggests that BMP–SMAD signaling may exert dual functions: on the one hand, promoting metastatic colonization and osteolysis in the bone microenvironment; on the other hand, facilitating immune surveillance and tumor suppression under specific conditions.

Mechanistically, the BMP–SMAD axis also interacts with multiple oncogenic pathways implicated in bone metastasis, including ER signaling, PI3K/AKT, and Hippo-YAP/TAZ networks. Crosstalk between these signaling modules can fine-tune SMAD transcriptional output, thereby modulating the balance between tumor progression and anti-tumor immunity. From a therapeutic perspective, targeting the BMP-SMAD pathway remains challenging due to its pleiotropic roles; systemic inhibition may disrupt physiological bone remodeling, whereas selective modulation of downstream effectors might offer a more precise strategy for preventing ER-positive breast cancer bone metastasis.

### 4.3. Hippo Signaling Pathway

The Hippo signaling pathway is a key regulator of tissue homeostasis, organ size, and tumorigenesis. In the context of breast cancer, particularly ER-positive subtypes with bone-metastatic potential, dysregulation of Hippo signaling plays a pivotal role in promoting EMT, stemness, invasion, and niche adaptation. The central effectors of this pathway, Yes-associated protein (YAP) and transcriptional coactivator with PDZ-binding motif (TAZ), are transcriptional coactivators that, when dephosphorylated, translocate into the nucleus to drive oncogenic gene expression through interaction with TEAD transcription factors [71,72].

Suppression of Hippo signaling and EMT activation. One major mechanism by which Hippo signaling is suppressed in breast cancer involves activation of the coagulation factor II receptor (F2R, also known as PAR1). F2R signaling inactivates the upstream MST1/2-LATS1/2 kinase cascade, resulting in decreased phosphorylation of YAP/TAZ and their nuclear accumulation [73,74]. In the nucleus, YAP/TAZ promote EMT by inducing transcriptional programs that enhance stem-like properties, motility, and resistance to apoptosis-hallmarks of metastatic competence.

Crosstalk with TGF-β and mechanotransduction. YAP/TAZ also interact functionally with the TGF β signaling axis, reinforcing metastatic signaling loops. For example, the YAP-TEAD complex upregulates thrombospondin-1 (TSP1), a TGF β activator that also stimulates FAK. FAK activation enhances cell–matrix adhesion and cytoskeletal remodeling, facilitating breast cancer cell invasion and migration [75]. This mechanochemical crosstalk is further intensified in stiff, hypoxic microenvironments such as bone.

Modulation by hypoxia and COX-2 inhibition. In hypoxic tumor niches, YAP/TAZ activity is sustained through HIF-1α and Snail upregulation, which inhibit LATS1/2 kinases and maintain YAP/TAZ in an active state. However, pharmacologic COX-2 inhibition downregulates HIF-1α and Snail, reactivating LATS kinases, leading to TAZ phosphorylation, cytoplasmic sequestration, and reduced oncogenic transcription [76]. These findings suggest the hypoxia-COX-2-Hippo axis as a potential therapeutic target in bone metastasis.

Integration with Wnt/β-catenin and RANKL signaling. In the bone microenvironment, Wnt pathway activation diminishes β-catenin degradation, allowing nuclear β-catenin to cooperate with YAP/TAZ–TEAD complexes. This interaction promotes expression of genes that support bone colonization, such as those related to osteomimicry, matrix remodeling, and immune evasion [77,78]. Furthermore, Hippo signaling regulates osteoclastogenesis via transcriptional control of connective tissue growth factor and modulation of TRAF6-mediated RANKL/RANK signaling, influencing key downstream cascades including NFκB, JNK, MAPK, and calcium signaling, which collectively enhance osteoclast activity and bone resorption [5]. Endogenous suppression and therapeutic implications. Recent studies have identified Dystonin (DST) as a negative regulator of YAP. DST expression promotes YAP inactivation and inhibits tumor progression and metastasis, underscoring the therapeutic potential of restoring or mimicking Hippo pathway activity [79]. Given the extensive crosstalk between Hippo signaling and multiple oncogenic pathways (e.g., TGF-β, Wnt/β-catenin, RANKL), its regulatory influence spans across EMT induction, immune modulation, metastatic dormancy escape, and skeletal colonization.

In summary, the Hippo pathway operates as a central node integrating mechanical, inflammatory, hormonal, and developmental cues to govern breast cancer cell plasticity and metastatic fitness. Its dynamic interactions with the bone microenvironment and other signaling cascades make it a compelling target for interventions aimed at mitigating bone metastasis in ER-positive breast cancer.

### 4.4. ERα-Src-p190 RhoGAP Signaling Pathway

The estrogen receptor alpha (ERα) -Src-p190 RhoGAP signaling axis plays a pivotal role in modulating breast cancer cell behavior within the bone microenvironment and has been increasingly recognized as a critical driver of skeletal metastasis, particularly in ER-positive subtypes. This pathway orchestrates a complex interplay between hormonal signaling and cytoskeletal regulation, facilitating tumor cell proliferation, survival, and colonization in bone.

Src, a non-receptor tyrosine kinase and member of the Src family kinases (SFKs), is a well-established mediator of osteolysis and osteoclast activation—two hallmark processes of breast cancer bone metastasis. Elevated Src activity has been closely associated with late-onset bone metastasis and correlates with increased bone turnover and poor prognosis in breast cancer patients [80,81]. Preclinical and early-phase clinical studies have demonstrated the therapeutic potential of Src inhibitors such as dasatinib and bosutinib in limiting both primary tumor growth and metastatic dissemination to the bone [82,83]. At the molecular level, Src acts as a signaling integrator, linking ER signaling to multiple downstream cascades, including the PI3K/AKT, MAPK, and cytoskeletal remodeling pathways [84,85]. ERα can form ligand-dependent or -independent complexes with Src, resulting in its activation. One of Src’s key substrates is p190 RhoGAP (ARHGAP35), a Rho GTPase-activating protein that negatively regulates RhoA signaling. Src-mediated phosphorylation of p190 RhoGAP at tyrosine 1105 (Y1105) enhances its GTPase activity, leading to inactivation of RhoA and downstream cytoskeletal reorganization [86,87]. This promotes reduced cell contractility and motility, favoring a switch from migratory to proliferative phenotypes-an adaptive advantage for disseminated tumor cells (DTCs) establishing residence within the bone niche.

Importantly, this pathway does not act in isolation. The ERα-Src-p190 RhoGAP axis interacts with multiple signaling modules involved in mechanotransduction, immune evasion, and dormancy escape, all of which contribute to the dynamic regulation of metastatic progression. Moreover, the role of Src in both tumor and bone-residing stromal cells, including osteoclasts and osteoblasts, further underscores its dual compartmental influence in shaping a metastasis-permissive microenvironment.

Taken together, the ERα-Src-p190 RhoGAP signaling cascade exemplifies a tightly regulated, context-dependent network that integrates hormonal cues with mechanical and structural signaling processes. Its capacity to regulate both proliferative signaling and cytoskeletal plasticity positions it as a promising therapeutic target in the treatment of ER-positive breast cancer bone metastasis. However, the context-specificity of Src inhibition across different metastatic organs necessitates further investigation into its organotropism and therapeutic window in clinical applications.

### 4.5. PAK4/Cyclin D1 Signaling Pathway

In ER^+^ breast cancer, estrogen regulates multiple signaling pathways that contribute to tumor growth, metastasis, and therapeutic resistance. Among these, the estrogen (17β-estradiol, E2)-mediated PAK4/Cyclin D1 axis has emerged as a key player in the progression of bone metastasis.

Studies have shown that E2 stimulation significantly increases the expression and activation of PAK4 in ER^+^ MCF-7 breast cancer cells [88]. Interestingly, PAK4 is closely associated with the activity of cancer stem-like cells (CSCs) in metastatic ER^+^ breast cancer. These CSCs tend to accumulate following anti-estrogen treatments, suggesting that PAK4 may contribute to endocrine therapy resistance [89]. Furthermore, PAK4-driven cell proliferation has been linked to activation of the PAK4/c-Src/EGFR/Cyclin D1 signaling cascade [90,91]. Multiple studies have confirmed that PAK4 promotes both gene and protein expression of Cyclin D1 [92], a well-established marker of poor prognosis in invasive breast cancer [93]. However, the direct molecular mechanisms through which PAK4 regulates Cyclin D1 remain incompletely understood.

Cyclin D1, a member of the D-type cyclin family (which also includes Cyclins D2 and D3), shares similar functions in cell cycle control. Cyclin D1 is uniquely overexpressed in solid tumors and promotes cancer progression through transcriptional regulation, maintenance of chromosomal stability, and modulation of cell migration. Although the CCND1 gene does not contain a canonical ERE in its promoter, its transcriptional activation is likely mediated by ER through interaction with a cAMP response element-binding site [94]. Functionally, Cyclin D1 acts as a mitogenic sensor by forming active complexes with cyclin-dependent kinases 4 and 6 (CDK4/6), driving the transition from G1 to S phase of the cell cycle. This pathway leads to phosphorylation of retinoblastoma protein (pRb), the release of E2F transcription factors, and subsequent expression of cell cycle–promoting genes [95,96]. Notably, Cyclin D1 has also been shown to induce secretion of osteopontin (OPN) in breast epithelial cells, a factor capable of promoting the expansion of bone marrow–derived stem cells, thereby potentially enhancing tumor adaptation and colonization within the bone microenvironment [97].

In summary, the E2/PAK4/Cyclin D1 signaling axis plays a pivotal role in regulating proliferation, cancer stemness, and bone-specific metastatic behavior in ER^+^ breast cancer. These findings not only deepen our understanding of estrogen-driven oncogenic pathways and resistance mechanisms but also highlight potential therapeutic targets for managing advanced diseases.

### 4.6. CDK4/6 Signaling Pathway

Cyclin-dependent kinases (CDKs) are a family of serine/threonine kinases that regulate cell cycle progression by partnering with their corresponding cyclins. Among them, CDK4 and CDK6—which share approximately 71% amino acid sequence identity—form complexes with D-type cyclins (D1, D2, D3) to drive the transition from the G1 phase to the S phase of the cell cycle. This is achieved through phosphorylation of the retinoblastoma protein (Rb), a critical tumor suppressor that normally inhibits E2F transcription factors to restrict entry into the S phase. Upon phosphorylation by CDK4/6, Rb becomes inactivated, freeing E2F to promote the transcription of genes involved in DNA synthesis and cell proliferation [98].

In luminal ER^+^ breast cancer, CDK4/6 activity is a major driver of malignancy and is closely linked to ER signaling [99]. E2 binds to ER, promoting receptor dimerization and nuclear translocation, where it directly regulates transcription of downstream genes by binding to EREs in their promoters [100]. One of the key ER target genes is CCND1, encoding Cyclin D1, which is consistently overexpressed in ER^+^ breast cancers and plays a central role in maintaining sustained CDK4/6 activation. Notably, Cyclin D1 has also been shown to activate ER signaling in a ligand-independent manner, suggesting a mechanism for endocrine resistance. Furthermore, E2 stimulation has been reported to upregulate D-type cyclins and enhance CDK4/6 activity in osteoblasts, indicating a potential role in promoting a pro-metastatic bone microenvironment [101].

CDK4/6 Inhibitors in ER^+^ Breast Cancer with Bone Metastasis. In recent years, three small-molecule inhibitors targeting dual CDK4/6-Palbociclib (PD-0332991), Ribociclib (LEE011), and Abemaciclib (LY-2835219)-have been approved by the U.S. Food and Drug Administration (FDA). These agents have demonstrated notable clinical efficacy, particularly when used in combination with endocrine therapy for the treatment of patients with ER^+^ breast cancer exhibiting extensive bone metastases [102,103]. Phase III clinical trials for all three CDK4/6 inhibitors have been completed, and results consistently show that the addition of CDK4/6 inhibitors to endocrine therapy significantly prolongs progression-free survival (PFS) compared to endocrine therapy alone in patients with advanced ER^+^ breast cancer [104]. This therapeutic combination has therefore become a standard of care in the management of hormone receptor-positive metastatic breast cancer.

However, despite initial efficacy, resistance to endocrine therapy, either intrinsic or acquired, remains a major clinical challenge. Up to 25% of ER^+^ breast cancer patients relapse within 10 years of treatment [105,106,107]. Dysregulation of the CDK4/6-Rb pathway has been implicated as a key contributor to therapeutic resistance. Emerging evidence suggests that CDK4/6 inhibitors may enhance radiosensitivity and improve outcomes when combined with radiotherapy, potentially through suppression of downstream proliferative and survival pathways such as ERK and NF-κB/c-Myc [108].

Furthermore, preclinical studies indicate that CDK4/6 inhibitors exert suppressive effects on osteoclast differentiation and downregulate the expression of bone resorption markers, suggesting a protective role in the bone microenvironment. Importantly, these agents do not impair osteoblast function or bone matrix formation, thereby preserving bone integrity in the context of bone-metastatic breast cancer [109].

### 4.7. Key Proteins Modulating Signaling Pathways in Breast Cancer Bone Metastasis

Breast cancer bone metastasis is governed by a complex interplay of signaling networks and transcriptional programs. In ER^+^ breast cancer, several key proteins serve as critical modulators of these pathways, promoting both tumor progression and adaptation to the bone microenvironment.

One of the most pivotal regulators is Runx2 (Runt-related transcription factor 2), a lineage-specific transcription factor essential for osteogenesis and increasingly recognized for its role in cancer metastasis [110]. In breast cancer cells, Runx2 facilitates EMT by repressing E-cadherin, upregulating vimentin, and enhancing cellular invasiveness. It also transcriptionally activates SNAI2 (Slug), an EMT-inducing factor normally degraded by the ubiquitin–proteasome system. Through this mechanism, Runx2 enhances cancer stem cell traits, promotes survival in hostile environments, and supports metastatic dissemination [111,112,113,114]. Runx2-driven SNAI2 expression is mediated through activation of the Wnt/β-catenin and TGF-β signaling pathways. Importantly, E2 has been shown to antagonize Runx2-mediated transcriptional activity, revealing a critical axis of crosstalk between hormonal signaling and osteogenic transcriptional networks. Interestingly, breast cancer cells that metastasize to bone often maintain high levels of both Runx2 and SNAI2, even in the context of elevated ERα expression, highlighting a distinct molecular signature of bone tropism in ER^+^ disease [115].

Another key signaling module implicated in bone metastasis is the mitogen-activated protein kinase (MAPK) pathway. Activation of MAPK cascades (e.g., ERK1/2) stimulates the transcription factors c-Fos and c-Jun, forming the AP-1 complex, which drives proliferation-associated gene expression. In ER^+^ breast cancer, loss of SLIT2, a known tumor suppressor, has been linked to hyperactivation of the MAPK/c-Fos axis, facilitating increased metastatic potential [116]. Notably, p38 MAPK exhibits dual functionality—acting either as a tumor suppressor or a promoter depending on cellular and microenvironmental context [117,118]. Further research is required to define the context-dependent behavior of MAPK subtypes, especially in hormone-responsive metastatic settings.

Beyond transcription factors and kinases, the tumor–bone microenvironment interaction is orchestrated by reciprocal signaling among osteocytes, mesenchymal stem cells (MSCs), and tumor cells. Deregulation of pathways including Wnt/β-catenin (e.g., LRP5, β-catenin overexpression) and PI3K/Akt has been shown to promote tumor cell survival and adaptation in the bone niche [119,120]. Within this framework, the EMT regulator SNAIL (SNAI1) emerges as a key node. SNAIL promotes resistance to apoptosis and genotoxic stress and can be transcriptionally induced by ER, particularly under 4-hydroxytamoxifen (4-OHT) stimulation via direct recruitment to the SNAI1 promoter. Paradoxically, SNAIL has also been observed to inhibit proliferation in certain contexts, such as invasive lobular carcinoma, underscoring its context-dependent and hormone-independent roles in tumorigenesis [121].

Collectively, these regulatory proteins and signaling circuits shape the molecular landscape of breast cancer bone metastasis in ER^+^ disease. They not only modulate intrinsic tumor behavior but also mediate adaptation to the unique pressures of the bone microenvironment. Understanding the functional integration of these pathways provides a basis for identifying novel therapeutic targets and refining strategies for preventing and treating skeletal metastases.

## 5. The Bone Microenvironment: A Fertile Soil for Breast Cancer Seeding and Growth

Breast cancer metastasis to bone is a highly orchestrated, multistep process involving local invasion, intravasation, circulatory survival and arrest, extravasation, and ultimately colonization and outgrowth at the secondary site [122]. Among distant organs, the bone microenvironment represents a particularly permissive and supportive niche for metastatic seeding and expansion of ER^+^ breast cancer cells. The successful establishment of bone metastases depends not only on the intrinsic properties of tumor cells but also on their ability to interact with and co-opt the complex cellular and molecular components of the bone milieu [123]. This microenvironment comprises osteoblasts, osteoclasts, bone marrow-derived immune cells, mesenchymal stromal cells, and the extracellular matrix (ECM)—all of which contribute to a dynamic signaling network.

By integrating signals from diverse cellular components, the bone microenvironment fosters a pro-metastatic niche that supports the colonization and outgrowth of breast cancer cells. This specialized niche enhances tumor cell survival by providing growth factors and anti-apoptotic signals, facilitates immune evasion through the suppression of cytotoxic immune responses, and promotes active remodeling of the bone matrix, releasing cytokines such as TGF-β and IGF-1 that further drive tumor proliferation. Moreover, a reciprocal crosstalk is established, wherein cancer cells stimulate osteoclast-mediated bone resorption, which in turn creates space and releases factors that support tumor expansion—a phenomenon referred to as the “vicious cycle” of bone metastasis. The following sections will examine in detail the cellular and molecular mechanisms by which the bone microenvironment is remodeled and co-opted to promote breast cancer progression.

In ER^+^ breast cancer, YAP acts as a mechanosensitive transcriptional co-regulator that links extracellular mechanical cues to changes in ER signaling, cytoskeletal organization and cell-cycle control. Higher tumor stiffness correlates with nuclear YAP1 accumulation [124]. Mechanistically, stiff ECM activates integrin/FAK and Rho GTPases, increases actomyosin tension and nuclear deformation, and thereby promotes YAP/TAZ nuclear localization and transcriptional activity. YAP/TAZ-TEAD complexes induce genes involved in actin cytoskeleton remodeling, focal adhesions, and ECM production, creating a feed-forward loop that stiffens the pericellular matrix and sustains high cell contractility [125]. This alters mechanotransduction by resetting how cells sense and respond to tension, which favors survival under mechanical stress and supports invasive behavior. Models overexpressing ERα36-mediated proliferation link membrane estrogen signaling to YAP-dependent cytoskeletal and proliferative pathways [126]. Pl3K and other oncogenic pathways can positively regulate YAP/TAZ in mammary cells, reinforcing mechanosensitive survival and growth signaling downstream of ER and growth factor receptors. By repressing ESR1 and modulating the TEAD-ERα axis, Hippo/YAP signaling can shift cells from classic ER-driven proliferation to YAP-centric, mechano-responsive growth programs. Small-molecule or epigenetic modulators of Hippo/YAP can reduce ESR1 expression or YAP activity suppress growth of ER^+^ cell lines and patient-derived organoids [127]. These highlight that mechanotransduction via YAP is both a driver and a potential vulnerability in ER^+^ breast cancer.

### 5.1. Stromal Cells

Stromal cells within the tumor microenvironment, particularly those located at the tumor–stroma interface, play a pivotal role in modulating breast cancer progression and metastasis. Transcriptomic profiling of tumor biopsies has revealed that genes regulating cell cycle progression are significantly enriched at this interface and closely associated with disease advancement [128]. Notably, epithelial–EMT markers often appear earliest in cancer cells adjacent to the stromal boundary. Stromal fibroblasts residing at the interface exhibit a stronger capacity to induce EMT than those located in surrounding, non-interface regions [129,130].

Among matrix-associated factors, OPN has emerged as a prominent biomarker in breast cancer [131]. Elevated OPN expression is associated with poor clinical outcomes, as higher levels correlate with reduced patient survival [132]. Interestingly, OPN exhibits context-dependent functionality: in tumor cells, it promotes tumorigenesis and survival in circulation, whereas in Lrp5-overexpressing osteoblasts, OPN may exert anti-proliferative effects on tumor cells [133]. Furthermore, OPN derived from bone marrow stromal cells contributes to the immunosuppressive characteristics of the metastatic niche, supporting cancer cell colonization and persistence [134].

The functional phenotype of breast cancer cells is profoundly influenced by stromal interactions within the bone marrow microenvironment, which directly impacts disease trajectory and therapeutic response [135]. MSCs, key constituents of the stromal compartment, possess multilineage differentiation potential and are instrumental in enhancing the invasiveness of ER^+^ breast cancer cells. In the bone marrow niche, MSCs facilitate the reactivation of dormant tumor cells and establish a milieu conducive to metastasis [136,137]. Intercellular communication is central to this process. Structures such as tunneling nanotubes (TNTs) and gap junctions, primarily mediated by connexin 43 (Cx43), facilitate the direct exchange of signals and biomolecules between stromal and tumor cells. Breast cancer cells upregulate both Cx43 expression and gap junction formation, enhancing molecular crosstalk with MSCs [138]. Through these conduits, tumor cells acquire growth factors, signaling proteins, and components of pathways such as PI3K/Akt, which further promote TNT formation and intercellular transport. This interaction stimulates downstream signaling events, including VEGF activation, thereby supporting angiogenesis and tumor expansion [139]. The dynamic interplay among tumor, stromal, and immune cells within the bone marrow niche establishes a permissive environment that promotes metabolic adaptation, immune evasion, and long-term colonization [140]. Targeting intercellular communication networks such as TNTs and gap junctions holds promise as a novel therapeutic strategy to disrupt breast cancer bone metastasis and reduce the risk of disease recurrence.

### 5.2. Osteoclasts and Osteoblasts

**Figure 4:**
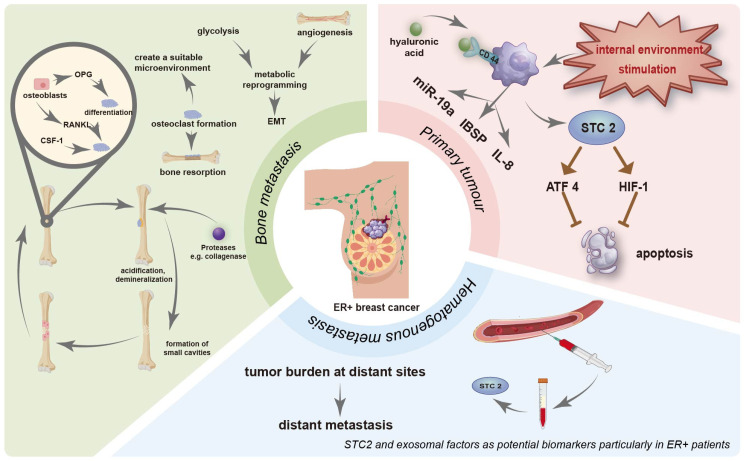
The steps that bone metastasis of ER^+^ breast cancer works. While mechanisms shown in green represent general bone metastatic mechanisms that are common to different breast cancer subtypes., the pink and blue panels highlight mechanisms that have been specifically validated in ER^+^ breast cancer cells.

The preferential colonization of bone by breast cancer cells is largely driven by the activity of bone-resorbing osteoclasts, which are activated by two key factors secreted by osteoblasts: receptor activator of RANKL and macrophage colony-stimulating factor (CSF-1) [141]. This activation process acidifies the local microenvironment and stimulates the release of proteolytic enzymes such as collagenase, resulting in bone demineralization and the formation of resorptive lacunae. These cavities are subsequently filled by newly recruited osteoblasts, thereby perpetuating a vicious cycle of bone remodeling and tumor growth. To regulate osteoclast activity, osteoblasts also secrete osteoprotegerin (OPG), a soluble decoy receptor that binds to RANKL, thereby preventing excessive osteoclast activation and maintaining bone homeostasis [142] (Figure 4). However, in ER-positive breast cancer, tumor cells can disrupt this balance by inducing osteoclastogenesis through various mechanisms. These include the upregulation of IBSP and the secretion of extracellular vehicles (EVs) enriched with exosomal miR-19a, both of which promote osteoclast differentiation and bone resorption [12]. Additionally, cytokine-mediated signaling enhances the interaction between cancer cells and the bone metastatic niche, fostering a pro-metastatic microenvironment [143]. The bone microenvironment not only supports cancer cell colonization but also actively influences tumor plasticity. For instance, epigenetic reprogramming driven by EZH2 within the bone niche alters the phenotype of ER-positive breast cancer cells, promoting endocrine therapy resistance and impacting the effectiveness of late-stage treatments [144].

Osteocytes, the most abundant cells in bone tissue, also contribute to this process. They interact with metastatic breast cancer cells through direct cell-to-cell contact and through the secretion of soluble mediators that promote osteoclast activation [145,146,147]. A critical cytokine in this process is interleukin-8 (IL-8/CXCL8), which is commonly overexpressed in primary breast tumors and can stimulate both osteoclastogenesis and bone resorption, independent of other tumor-derived signals [148]. IL-8 not only enhances the invasiveness and survival of circulating tumor cells (CTCs) but also promotes bone tropism and colonization in breast cancer. Notably, anti-IL-8 therapy has been shown to effectively suppress tumor colonization, growth, and bone destruction, highlighting IL-8 as a potential therapeutic target in bone-metastatic breast cancer [149].

### 5.3. Metastasis-Related Proteins

Hypoxia is a hallmark of the tumor microenvironment and a critical driver of breast cancer aggressiveness. Under hypoxic conditions, hypoxia-inducible factors (HIFs), particularly HIF-1α, become stabilized and transcriptionally activated under hypoxic conditions, initiating the transcription of target genes involved in angiogenesis, EMT, and glycolysis-based metabolic reprogramming [150]. Through these coordinated responses, HIF-1α promotes tumor cell survival, proliferation, and metastatic dissemination, ultimately contributing to the development of an aggressive breast cancer phenotype [53].

Hyaluronic acid (HA) is another critical factor in metastasis. Elevated levels of circulating HA have been shown to inhibit tumor metastasis, largely due to their ability to disrupt interactions between tumor cells and vascular endothelial cells [151]. However, this effect is counteracted by the presence of CD44, the principal receptor for HA. CD44 is a transmembrane glycoprotein that is frequently expressed on stem-like breast cancer cells, which can be detected in the bone marrow of patients with early-stage breast cancer [152]. CD44 facilitates adhesion to bone marrow endothelium, enhances the activity of collagen-degrading enzymes, and initiates matrix degradation, particularly of collagen I-rich bone tissue [153]. Elevated CD44 expression correlates with increased tumor burden, enhanced distant metastasis, and reduced survival rates, providing clinical evidence that CD44-positive breast cancers are more prone to recurrence and distant spread. Experimental models further confirm that the absence of CD44 weakens tumor-endothelial adhesion and reduces invasiveness, though not affecting proliferation, while injection of CD44-positive cells promotes bone metastasis and myeloma-like lesions in vivo [154].

Another metastasis-associated protein is Stanniocalcin 2 (STC2), a glycosylated secretory protein that is detectable in serum and considered a potential biomarker for cancer prognosis. STC2 contributes to multiple aspects of tumor biology, including proliferation, invasion, and metastatic spread [155]. It also participates in stress response pathways that are evolutionarily conserved. Under endoplasmic reticulum stress, hypoxia, or nutrient deprivation, STC2 is upregulated and activates key survival regulators such as activating transcription factor 4 (ATF4) and HIF-1, thereby inhibiting apoptosis and enhancing tumor cell adaptation [156,157]. In breast cancer, STC2 expression is positively correlated with ER status, suggesting potential relevance to ER-positive subtypes [158]. However, whether STC2 levels can serve as a reliable prognostic marker remains to be clarified, and further research is needed to determine their clinical utility [159,160]. Collectively, these findings underscore the potential of HIF-1α, CD44, and STC2 as both biomarkers and therapeutic targets in the management of breast cancer bone metastasis.

In summary, the bone microenvironment actively promotes ER-positive breast cancer metastasis by supporting tumor survival, immune evasion, and therapeutic resistance. Understanding these interactions lays the groundwork for targeted treatment. The next section discusses current therapeutic strategies and predictive approaches to manage bone metastases more effectively.

## 6. Prediction and Treatment

As breast cancer remains a central focus of oncologic research, a growing number of therapeutic agents targeting bone metastasis have been developed. These treatments primarily aim to inhibit tumor proliferation and metastatic progression by modulating genetic regulators, signaling pathways, and the tumor microenvironment. The overarching therapeutic goals include controlling tumor growth, preventing bone colonization, and, importantly, reducing the incidence of skeletal-related events (SREs), which significantly impact patient quality of life and survival outcomes.

### 6.1. Predictive Biomarkers for Bone Metastasis

In addition to therapeutic strategies, early detection of bone metastasis is critical for improving clinical outcomes in breast cancer patients. Identification of reliable predictive biomarkers is essential for the timely recognition of patients at high risk of skeletal dissemination. One promising biomarker is the breast osteoblast-like cell (BOLC), a unique cell population within breast tissue that co-expresses RUNX2 and RANKL. These cells possess osteoblastic properties and are capable of producing calcified hydroxyapatite crystals [161]. The presence of such crystals often manifests as casting-type calcifications on mammographic imaging and is closely associated with malignant transformation and poor prognosis [162].

Accumulating evidence suggests that BOLCs are positively correlated with the likelihood of bone metastasis in primary breast tumors. Specifically, their abundance is associated with increased expression of EMT markers such as TGF-β and vimentin and shows a strong linear correlation with ER-positive tumor cells. Interestingly, research by Manuel et al. highlighted an inverse relationship between the presence of BOLCs and the maintenance of breast cancer stem cells, suggesting a complex interplay between tumor cell differentiation and metastatic potential [163]. Immunohistochemical analysis has also identified other markers predictive of bone metastasis risk. Tumors from patients with skeletal metastases often exhibit elevated expression of CD44 and trefoil factor 1 (TFF-1), compared to non-metastatic cases [164]. These findings underscore the potential of incorporating histopathological biomarkers into risk stratification models to guide individualized surveillance and early intervention strategies. Driven by scientific and technological advancements, these molecular processes can be detected by atomic force microscopy (AFM) technologies and positron emission tomography (PET) in order to contribute to diagnostic accuracy and refine treatment approaches [165,166].

### 6.2. Endocrine Therapy Improves ER-Positive Tumors

A cross-sectional study conducted in China demonstrated that endocrine therapy significantly improves the prognosis of patients with ER-positive metastatic breast cancer [167]. Currently, endocrine therapy remains the cornerstone of treatment for ER-positive breast cancer. ERα, a member of the nuclear receptor transcription factor family, plays a central role in the initiation and progression of ER^+^ tumors. Therefore, therapeutic strategies primarily focus on inhibiting ERα signaling activity. The most commonly used endocrine therapies include tamoxifen, a sSERM, and aromatase inhibitors, both of which effectively block estrogen signaling in tumor cells. These agents are often administered in combination with CDK4/6 inhibitors, such as palbociclib, which has been shown to prolong PFS and overall survival (OS) in patients with advanced ER-positive disease [168]. In recent years, lasofoxifene, a third-generation nonsteroidal SERM originally developed for postmenopausal osteoporosis, has shown enhanced antitumor efficacy compared to fulvestrant. It is more effective in inhibiting primary tumor growth and reducing metastatic burden, particularly in preclinical models. Notably, the combination of lasofoxifene with palbociclib demonstrates synergistic effects in suppressing bone tumor growth and preventing metastasis [103]. However, the combination of palbociclib with letrozole or fulvestrant has not consistently shown a significant advantage over monotherapy with either letrozole or fulvestrant in terms of PFS and OS in some clinical settings, and the underlying mechanisms for this limited benefit remain unclear, warranting further investigation [169].

### 6.3. Gene-Related Drugs

Paclitaxel has long been considered a cornerstone chemotherapeutic agent in the treatment of various cancers, including ER-positive breast cancer. However, the emergence of drug resistance significantly limits its long-term efficacy. Key genes implicated in paclitaxel resistance include estrogen receptor 1 (ESR1), cyclin D1 (CCND1), and SCUBE2. Among them, SCUBE2 has emerged as a promising predictive biomarker for both ER-positive/HER2-negative and HER2-positive breast cancers, with predictive capabilities comparable to those of ESR1 [170]. In addition to chemotherapy resistance, therapeutic strategies have been developed to address bone loss and osteolytic damage associated with breast cancer bone metastasis. One such approach involves the use of adeno-associated virus (AAV) vectors engineered to express OPG. Studies have shown that this treatment not only reverses bone loss and polyethylene particle-induced osteolysis but also preserves endogenous OPG levels without adverse effects [171]. Notably, recombinant AAV-OPG (rAAV-OPG) therapy significantly enhances trabecular bone volume and connectivity density, thereby promoting bone repair and reducing the structural fragility associated with metastasis-induced bone lesions. Moreover, this gene therapy approach does not exhibit systemic toxicity. Although minor hematological changes such as red blood cell fragmentation, elevated reticulocyte counts, and increased platelet levels have been observed, these effects are within tolerable limits. Overall, rAAV-OPG gene therapy shows substantial potential in mitigating osteolytic complications and improving clinical outcomes for breast cancer patients with bone metastases [172].

### 6.4. Signaling Pathway-Related Drugs

Recent studies have identified a variety of drugs that target key components of signaling pathways involved in breast cancer bone metastasis. By interfering with specific molecular nodes, these agents inhibit cancer cell proliferation, invasion, and colonization within the bone microenvironment (Table 2). CDK4/6 inhibitors, including palbociclib, ribociclib, and abemaciclib, in combination with endocrine agents like fulvestrant, have demonstrated clinical efficacy and safety in diverse populations, including Asian patients [173]. Since their FDA approval in 2015, CDK4/6 inhibitors have become a standard first-line treatment for hormone receptor–positive advanced breast cancer worldwide. Kaempferol, a naturally occurring flavonoid found in fruits and vegetables, exhibits broad-spectrum antitumor activity. It suppresses breast cancer progression by downregulating EMT-related markers and inhibiting the PI3K/Akt signaling pathway, thereby impeding both metastasis and therapeutic resistance [174]. PERK (protein kinase RNA-like endoplasmic reticulum kinase), which is highly expressed during integrin-mediated stress responses in breast cancer cells, has emerged as a novel target. Inhibiting PERK has been shown to reduce ectopic metastasis, suggesting potential for preventing secondary tumor spread [175]. In addition, ononin, an isoflavone compound, has demonstrated antitumor potential by blocking MAPK pathway phosphorylation, inhibiting osteolytic factor production, and reversing EMT phenotypes. These mechanisms support its promise as a therapeutic candidate for breast cancer bone metastasis [176]. Given the complexity of signaling networks in metastatic progression, pathway-specific interventions represent a critical avenue for precision therapy in advanced ER-positive breast cancer.

### 6.5. Tumor Microenvironment-Related Drugs

The bone microenvironment plays a critical role in facilitating the colonization and progression of breast cancer cells, primarily through the action of various cytokines, stromal cells, and osteoclast-mediated bone remodeling. Accordingly, therapeutic strategies aimed at modulating the tumor microenvironment have garnered significant attention.

Denosumab, a fully human IgG2 monoclonal antibody targeting RANKL, effectively blocks the RANK-RANKL interaction, thereby inhibiting osteoclast differentiation and function. This suppresses bone resorption and has shown clinical benefit in preventing and delaying the progression of bone metastases in breast cancer patients [204]. Bisphosphonates, synthetic analogs of pyrophosphate that bind to hydroxyapatite in bone, also exert anti-resorptive effects by inducing osteoclast apoptosis. Intravenous administration of bisphosphonates is now widely used as a preventive measure against irreversible SREs in patients with advanced disease [205]. However, the efficacy of bisphosphonates as adjuvant therapy remains controversial. While some clinical trials have demonstrated improved outcomes, others report no significant benefit or even potential harm in certain patient subgroups [206]. Zoledronate, a potent nitrogen-containing bisphosphonate, has been shown to reduce the incidence of bone metastases in ER-positive breast cancer and may exert a protective effect by limiting the re-dissemination of circulating tumor cells to distant organs. Nonetheless, in premenopausal women with elevated estrogen levels and aggressive tumor phenotypes, zoledronate use has been associated with an increased risk of extra-skeletal metastasis, particularly to visceral organs [207]. Reversine, a synthetic purine derivative with multi-kinase inhibitory properties, has demonstrated anti-tumor activity in both hematologic and solid malignancies. Its therapeutic effects are partially mediated through modulation of the bone marrow stromal microenvironment [208]. Notably, reversine has been found to influence the late-stage growth of primary tumors by altering the expression of stromal genes, thereby reshaping the tumor-stroma interaction and contributing to tumor growth suppression [209].

### 6.6. Treatment of ER-Positive Breast Cancer Bone Metastasis with Chinese Herbal Medicine

Traditional Chinese medicine (TCM) has long played an integral role in the prevention and treatment of breast cancer bone metastasis (Table 3). Several natural compounds derived from herbal sources have demonstrated promising antitumor and bone-protective effects by targeting key signaling pathways involved in tumor progression and osteoclastogenesis.

Ailanthone (AIL), a quassinoid isolated from Ailanthus altissima, exerts potent anti-metastatic effects by upregulating the expression of FOXP3. This leads to the downregulation of multiple metastasis-associated signaling cascades, including RANKL, IL-1β, MAPK, PI3K/AKT, and NF-κB. Consequently, AIL suppresses the invasive and migratory capabilities of breast cancer cells while inhibiting osteoclast-related gene expression, thereby disrupting the formation of a metastatic niche in the bone microenvironment [216]. Curcumin, a polyphenol extracted from Curcuma longa, has been widely recognized for its low toxicity and broad-spectrum antitumor properties. It impairs the TGF-β/Smad signaling pathway, thereby reducing the expression of PTHrP, a key mediator of osteolytic bone destruction in breast cancer bone metastasis [217]. Punicalin, a polyphenolic compound found in Punica granatum, exhibits anti-resorptive activity by inhibiting the NF-κB signaling pathway. This results in suppression of osteoclast differentiation, F-actin ring formation, bone resorption, and the expression of osteoclast-specific genes. These effects collectively attenuate bone degradation and limit the extent of bone invasion by breast cancer cells [218]. In summary, these findings highlight the therapeutic potential of traditional Chinese herbal compounds as adjunctive agents in managing ER-positive breast cancer bone metastasis, offering multifaceted mechanisms to inhibit tumor progression and preserve bone integrity.

### 6.7. Chimeric Antigen Receptor-Engineered T (CAR-T) Therapies

Metastasis is a complex problem that hugely influences the prognosis of breast cancer. CAR-T cell therapy is a promising treatment method despite the lack of an immunosuppressive microenvironment and tumor-specific antigens. Therapeutic applicability is expanded by chemokine receptor engineering and promoting manufacturing processes with CAR platforms [221]. However, Claudia Arndt et al. showed that palbociclib can influence T cells while impacting the cell cycle of tumors, suggesting that patients undergoing T cell-based immunotherapy should take palbociclib cautiously or have a palbociclib-free period [222].

## 7. Conclusions and Expectation

Bone metastasis in breast cancer remains a critical and extensively investigated topic within oncology research. In this review, we recollected the history of discovery and development of bone metastasis in ER^+^ breast cancer and then examined the metastatic progression of breast cancer from three interconnected dimensions: genetic regulation, signaling pathways, and the tumor microenvironment. Rather than functioning independently, these aspects operate synergistically to drive the proliferation, invasion, metastatic spread, and colonization of breast cancer cells within the bone microenvironment.

Complex bidirectional interactions exist within this milieu. For instance, OPG promotes osteoclast differentiation, which in turn enhances osteoblast activity through Wnt/β-catenin signaling [223]. In breast cancer cells, the glycoprotein 130 (gp130) cytokine family activates multiple downstream pathways. Oncostatin M (OSM) exhibits tumor-promoting properties, whereas ciliary neurotrophic factor (CNTF) induces dormancy. Both factors exert their effects via gp130-mediated signaling through STAT3, ERK, and AKT. Elevated IL-6 levels in the bone microenvironment further potentiate STAT3 activation, contributing to tumor survival and dormancy. LIFR/STAT3 axis, expressed in breast cancer cells, has been implicated in maintaining dormancy within the bone niche [38]. In contrast, the loss of STAT3 signaling facilitates escape from dormancy, downregulates LIFR expression, and promotes tumor proliferation and colonization [39]. Furthermore, ER signaling exhibits tissue- and microenvironment-specific effects, modulated by cytokines, immune mediators, and stromal cells, which collectively contribute to endocrine resistance [224,225,226].

It is crucial to acknowledge the heterogeneity within ER-positive breast cancer. Subtypes such as ER^+^/PR^+^ and ER^+^/PR^−^ display distinct clinical characteristics. Patients with ER^+^/PR^+^ tumors typically experience the most favorable prognosis and survival outcomes, whereas those with ER^+^ but PR^−^ disease still tend to fare better than individuals with PR^−^ only expression [227]. These observations highlight the importance of accurate hormonal profiling and precise subtype classification to guide personalized therapeutic strategies.

By reviewing this field, we find comprehensive models that accurately recapitulate ER^+^ bone tropism, endocrine dependence, and late recurrence patterns remain limited. It is hard to show the key characteristics of bone metastasis in ER^+^ breast cancer, which are different from general osteolytic mechanisms. Due to difficulties in regularly obtaining samples from bone marrow and the long duration of metastasis, evidence of bone metastasis remains indirect and limited. The dormancy maintenance and escape of tumors call for more appropriate detection methods. Though we can detect the disease by AFM and PET, no biomarker has yet undergone rigorous, prospective validation in ER^+^ populations. Risk stratification for bone recurrence mainly relies on clinical variables rather than molecular models. In addition, there is significant space for development of its treatment. More pharmacology and clinical trials should be done to diminish the side-effects of treatment and improve bone-specific clinical trial design. Dedicated clinical trials are urgently needed to optimize the sequencing and combination of endocrine therapies, radiotherapy, SERDs, CDK4/6 inhibitors and bone-targeted agents. Advancing research along these lines will be critical for translating biological insights into improved prevention, risk stratification, and therapy.

In summary, breast cancer remains the most prevalent malignancy among women worldwide, with ER-positive tumors constituting a major subtype [228]. Bone metastasis in these patients involves a multifaceted interplay of signaling molecules, cellular interactions, and microenvironmental cues. Despite progress in therapeutic development, many challenges persist, including contradictory findings and dual-function molecules whose roles remain inadequately defined. Moving forward, a comprehensive, integrative approach that encompasses molecular, cellular, and systemic insights will be essential. Future studies should prioritize the identification of novel biomarkers, therapeutic targets, and rational combination regimens to improve early diagnosis, prevention, and treatment of bone metastases in ER-positive breast cancer.

## Figures and Tables

**Table 1:** The overall description of genes related to bone metastasis.

Classification	Genes	Character
Hormone-dependent genes	*SCUBE2*	Bridging ERα signaling and multiple metastasis-promoting pathways (HH, Notch, and immune evasion).
		Regulated by estrogen
		Contributing to both dormancy escape and bone niche adaptation
	*PAK4*	Overriding dormancy mechanisms via ERα interaction
		Promoting bone destruction through osteoclastogenic signaling
	*TOX3*	Acting as a context-specific regulator in ER-positive breast cancer
		Significant implications for disease progression and metastatic potential-particularly in skeletal colonization
	*NRIP1*	Serving as a critical regulatory node which connects estrogen pathway signaling with the genetic landscape of ER-positive breast cancer
		Influencing metastatic behavior (e.g., bone dissemination)
Hormone-independent genes	*TOR1B*	Helping tumors adapt to odious environment
	*PTHrP*	Promoting osteolytic bone metastasis
		Creating a fine microenvironment for cancer cell colonization

**Table 2:** The Clinical Status of protein kinase inhibitors.

Protein Kinase	Pathway	Function/Mechanism	Representative Inhibitors	Clinical Status	Potential Side-Effects (Main)
ERK5 (MAPK7) [177]	MAPK pathway branch	Related to bone microenvironment and cell motility	XMD8-92 (experimental)	Preclinical stage	To be studied
PERK	UPR–PERK–eIF2α	Tumor dormancy and survival under ER stress	GSK2656157, AMG-44	Preclinical to early clinical trials	To be studied
PAK4 [178]	PAK4–β-catenin/Wnt, EMT	EMT, stemness maintenance, bone metastasis, drug resistance	KPT-9274 (PAK4/NAMPT dual inhibitor)	Ongoing clinical trials (Phase I/II)	Anemia, kidney and stomach toxicity [179]
JAK/STAT3 [180]	IL-6–JAK–STAT3 axis	Tumor stemness, immune evasion, bone metastasis	Ruxolitinib (JAK1/2), Napabucasin (STAT3)	In clinical trials	Cytopenia (Ruxolitinib) [181], GI symptoms [182]
FAK (PTK2) [183]	Integrin–FAK–SRC–PI3K	Bone adhesion, invasion, metastasis	Defactinib, VS-6063	Clinical trials	Fatigue, GI symptoms [184]
CHK1/CHK2 [185]	DNA damage response	DNA repair, regulation of therapy resistance	Prexasertib (CHK1), AZD7762	Clinical trials	Leukopenia, neutropenia (Prexasertib) [186]; cardiotoxicity (AZD7762) [187]
SRC [188]	SRC–FAK–ERα axis	Bone metastasis, EMT, invasion, synergy with ER	Dasatinib, Bosutinib	Clinical trials; mixed outcomes	Pleural effusion, myelosuppression (Dasatinib) [189];diarrhea, hepatotoxicity (Bosutinib) [190]
MAPK/ERK [191]	RAS–RAF–MEK–ERK pathway	Proliferation, invasion, crosstalk with ER signaling	Trametinib (MEK), Selumetinib	Under evaluation for endocrine combination therapy	Dermatologic toxicity, GI symptoms [192,193]
CDK4/6 [194]	Cyclin D–CDK4/6–Rb	Cell cycle regulation; endocrine resistance	Palbociclib, Ribociclib, Abemaciclib	FDA approved for ER^+^ breast cancer	Neutropenia, nausea [195,196]
mTOR [197]	Downstream of PI3K–AKT–mTOR	Cell growth, metabolism, resistance	Everolimus, Temsirolimus	FDA approved (Everolimus with exemestane)	Stomatitis, GI symptoms [198,199]
PI3K/AKT/mTOR [200]	PI3K–AKT–mTOR pathway	Proliferation, anti-apoptosis, resistance, metabolic regulation	Alpelisib (PI3Kα), Everolimus (mTOR)	FDA approved (Alpelisib for PIK3CA mutations)	Stomatitis (Everolimus) [198]; Hyperglycemia, Gastrointestinal side effects (both) [201,202,203]

**Table 3:** The representative drugs for ER-Positive Breast Cancer Bone Metastasis.

Functional Route	Examples of Drug	Details
Endocrine therapy	tamoxifen [210,211]	ER modulators, mainly used in premenopausal patients, compete with estrogen for binding to ER and have the ability to mix agonists/antagonists.
	letrozole, anastrozole, exemestane [210,211]	Acting as aromatase inhibitors, they block the conversion of androgens to estrogens and reduce systemic estrogen levels in postmenopausal patients.
	fulvestrant [210,212,213]	ER downregulation works by inducing ER protein degradation or blocking ER transcriptional activity, and inhibiting ER activity by impairing ER migration in nuclear.
	neratinib [214]	HER2 tyrosine kinase inhibitor
	lasofoxifene [103]	They inhibit the growth of primary tumor cells and reduce metastasis
Genes	paclitaxel [170]	Efficient broad-spectrum anticancer drugs
Signaling pathway	palbociclib, ribociclib, abemaciclib [211]	CDK4/6 inhibitor
	alpelisib [201]	Specific inhibitors of PI3Kα
	everolimus [215]	mTORC1 inhibitor blocks a key signaling node downstream of PI3K
	kaempferol [174]	They downregulate EMT markers and PI3K/Akt signaling pathway.
	PERK inhibitor [175]	They prevent ectopic metastasis of breast cancer
	ononin [176]	They inhibit the phosphorylation of the MAPK signaling pathway, suppress the formation of osteolytic factors, and reverse epithelial mesenchymal transition markers.
	capivasertib [211]	AKT inhibitor
	ailantone [216]	They upregulate the expression of FOXP3 and inhibit the expression of various signaling pathways such as RANKL, IL-1β, MAPK, PI3K/AKT, and NF-κB
	curcuminoids [217]	They block the Smad signaling pathway which inhibits TGF-β to reduce PTHrP.
	punicalin [218]	NF-κB signaling pathway inhibitor
Tumor microenvironment	tocilizumab [219]	They inhibited the production of miR-221hi microvesicles by CAFs and the subsequent development of CAF-CSC niche by blocking IL-6.
	denosumab [204]	Anti-RANK-1 IgG2 antibody inhibits the activity of osteoclast.
	bisphosphonates [205]	They inhibit the activity of osteoclasts.
	zoledronate [207]	The drugs protect the re-dissemination of circulating tumor cells released from non-bone areas.
	reversine [208]	Anti-myeloma activity
	capecitabine, gemcitabine, fluorouracil, methotrexate [220]	Metabolic antagonist
	chlorogenic acid [12]	IBSPR inhibitor

## Data Availability

No new data were created or analyzed in this study. Data sharing is not applicable to this article.

## Abbreviations

The following abbreviations are used in this manuscript:

4-OHT	4-hydroxytamoxifen
AAV	adeno-associated virus
AIL	Ailanthone
ATF4	activating transcription factor 4
AIs	aromatase inhibitors
BMP	bone morphogenetic protein
BOLC	breast osteoblast-like cell
CCND1	cyclin D1
CDK4/6	cyclin-dependent kinases 4 and 6
ChIP	chromatin immunoprecipitation
CNTF	ciliary neurotrophic factor
CSCs	cancer stem-like cells
CSF-1	colony-stimulating factor
CTCs	circulating tumor cells
CTGF	connective tissue growth factor
Cx43	connexin 43
DST	Dystonin
DTCs	disseminated tumor cells
E2	estradiol
ECM	extracellular matrix
EMT	epithelial–mesenchymal transition
ER	Estrogen receptor
ERα	estrogen receptor alpha
ERβ	estrogen receptor beta
ER^+^	Estrogen receptor-positive
EREs	estrogen response elements
ESR1	estrogen receptor 1
ET-1	endothelin-1
EVs	extracellular vesicles
F2R	factor II receptor
FAK	focal adhesion kinase
FDA	the U.S. Food and Drug Administration
PERK	protein kinase RNA-like endoplasmic reticulum kinase
FGFs	fibroblast growth factors
HA	Hyaluronic acid
HER2	Human epidermal growth factor receptor 2
HH	Hedgehog
HIFs	hypoxia-inducible factors
IBSP	integrin-binding sialoprotein
IL-1	interleukin-1
IL-8	interleukin-8
IL-11	interleukin-11
LIFR	leukemia inhibitory factor receptor
MAPK	mitogen-activated protein kinase
MSCs	mesenchymal stem cells
MMPs	matrix metalloproteinases
NK	natural killer
NRIP1	Nuclear Receptor Interacting Protein 1
OPG	osteoprotegerin
OPN	osteopontin
OS	overall survival
OSM	Oncostatin M
PAK4	p21-activated kinase 4
PDGFs	platelet-derived growth factors
PFS	progression-free survival
PR	progesterone receptor
pRb	retinoblastoma protein
p-RUNX1	phosphorylated RUNX1
PTHrP	Parathyroid hormone-related protein
RANKL	receptor activator of nuclear factor kappa-B ligand
Rb	retinoblastoma protein
R-SMADs	receptor-regulated SMADs
Runx2	Runt-related transcription factor 2
SCUBE2	Signal peptide-CUB-EGF-like domain-containing protein 2
SERDs	selective estrogen receptor downregulators/degraders
SERMs	selective estrogen receptor modulators
SFKs	Src family kinases
SHH	Sonic Hedgehog
SREs	skeletal-related events
STC2	Stanniocalcin 2
TAZ	transcriptional coactivator with PDZ-binding motif
TCM	traditional Chinese medicine
TFF-1	trefoil factor 1
TGF-β	transforming growth factor-beta
TGFβRI	TGF-β receptor I
TGFβRII	TGF-β receptor II
TNTs	tunneling nanotubes
TOR1B	Torsin Family 1 Member B
TOX3	TOX High Mobility Group Box Family Member 3
TSP1	thrombospondin-1
YAP	Yes-associated protein
